# Transcriptome exploration of the sex pheromone gland of *Lutzomyia longipalpis* (Diptera: Psychodidae: Phlebotominae)

**DOI:** 10.1186/1756-3305-6-56

**Published:** 2013-03-07

**Authors:** Natalia González-Caballero, Jesus G Valenzuela, José MC Ribeiro, Patricia Cuervo, Reginaldo P Brazil

**Affiliations:** 1 Laboratório de Bioquímica e Fisiologia de Insetos, IOC, FIOCRUZ, Av. Brasil 4365, Manguinhos, Pav. Leônidas Deane, Sala 213, Rio de Janeiro, RJ, CEP: 21040-360, Brasil; 2 Laboratory of Malaria and Vector Research, NIAID, NIH, Rockville, MD, USA; 3 Laboratório de Pesquisa em Leishmaniose, IOC, FIOCRUZ, Rio de Janeiro, RJ, Brasil

**Keywords:** Lutzomyia longipalpis, Male pheromone gland, Transcriptome, Mevalonate pathway

## Abstract

**Background:**

Molecules involved in pheromone biosynthesis may represent alternative targets for insect population control. This may be particularly useful in managing the reproduction of *Lutzomyia longipalpis*, the main vector of the protozoan parasite *Leishmania infantum* in Latin America. Besides the chemical identity of the major components of the *L. longipalpis* sex pheromone, there is no information regarding the molecular biology behind its production. To understand this process, obtaining information on which genes are expressed in the pheromone gland is essential.

**Methods:**

In this study we used a transcriptomic approach to explore the pheromone gland and adjacent abdominal tergites in order to obtain substantial general sequence information. We used a laboratory-reared *L. longipalpis* (one spot, 9-Methyl GermacreneB) population, captured in Lapinha Cave, state of Minas Gerais, Brazil for this analysis.

**Results:**

From a total of 3,547 cDNA clones, 2,502 high quality sequences from the pheromone gland and adjacent tissues were obtained and assembled into 1,387 contigs. Through blast searches of public databases, a group of transcripts encoding proteins potentially involved in the production of terpenoid precursors were identified in the 4^th^ abdominal tergite, the segment containing the pheromone gland. Among them, protein-coding transcripts for four enzymes of the mevalonate pathway such as 3-hydroxyl-3-methyl glutaryl CoA reductase, phosphomevalonate kinase, diphosphomevalonate descarboxylase, and isopentenyl pyrophosphate isomerase were identified. Moreover, transcripts coding for farnesyl diphosphate synthase and NADP^+^ dependent farnesol dehydrogenase were also found in the same tergite. Additionally, genes potentially involved in pheromone transportation were identified from the three abdominal tergites analyzed.

**Conclusion:**

This study constitutes the first transcriptomic analysis exploring the repertoire of genes expressed in the tissue containing the *L. longipalpis* pheromone gland as well as the flanking tissues. Using a comparative approach, a set of molecules potentially present in the mevalonate pathway emerge as interesting subjects for further study regarding their association to pheromone biosynthesis. The sequences presented here may be used as a reference set for future research on pheromone production or other characteristics of pheromone communication in this insect. Moreover, some matches for transcripts of unknown function may provide fertile ground of an in-depth study of pheromone-gland specific molecules.

## Background

The sandfly *Lutzomyia longipalpis* (Lutz & Neiva, 1912) is the principal vector of *Leishmania infantum* (Protozoa: Kinetoplastida) in the Americas, a protozoan parasite responsible for American visceral leishmaniasis (AVL) [1-3]. *L. longipalpis* uses male-produced pheromones for mate recognition. Females perceive and respond to a specific chemical signal from distances of a few meters [4,5]. Additionally, there is evidence that the same pheromone also produces male aggregation [6]. These attractants are synthesized by specialized gland cells located beneath the cuticle in abdominal segments of adult insects [7]. Recently, a study with *L. longipalpis* obtained from Lapinha Cave, Minas Gerais, Brazil reported that pheromone biosynthesis started around 12 hours after emergence and increased for 3 days from then on, stabilizing thereafter [8]. Once the pheromone is produced, it is disseminated to the environment through papular structures located in pale abdominal spots [9]. In *L. longipalpis* populations these pale-spots phenotypes can be found as a single pair (1S) on the fourth abdominal segment, or as two pairs (2S), on both the third and fourth abdominal segments. The lighter-colored appearance of these segments, exposing the pheromone disseminating structure, is generally attributed to the absence of macrotrichias, widely distributed over the rest of the abdominal surface of the insect [10].

*Lutzomyia longipalpis* is apparently a complex of sibling species ([11], reviewed in [12]), although the number and distribution of potential component species remains to be established [13-15]. Early studies with different *L. longipalpis* populations identified the main chemical components of the pheromone blends belong to the terpene class of compounds [16-18]. Four distinct terpenes have been identified, two of which have been completely characterized to have 16 carbons (homosesquiterpenes) and two compounds having 20 carbons (diterpenes) of unknown stereochemistry. The homosesquiterpenes of the *L. longipalpis* sex phermone are (1S,3S,7R)-3-methyl-a-himachalene and (S)-9-methylgermacrene-B. In Brazil, these pheromones are produced by insects found in Jacobina, state of Bahia, and Lapinha Cave, state of Minas Gerais, respectively [17,19]. Other populations, such as *L. longipalpis* from Sobral, state of Ceará and Jaíba, state of Minas Gerais, produce two diterpene isomeres: cembrene1 and cembrene2, respectively [20]. A fifth pheromone-producing *L. longipalpis* population (chemotype) found in Sobral, state of Ceará, is distinguished for its varying amounts of terpenes, relative to the populations previously mentioned, as well as other morphological differences [16]. These diverse populations make the *L. longipalis* complex an interesting model for research on genetic regulation, enzymatic components, and pathways of pheromone production.

In addition to the *Lutozmyia* genus, there has been progress in the chemical communication research of other phlebotomine genera such as *Sergentomyia* and *Phlebotomus*[21]. Although terpenes similar to those of the *L. longipalpis* species complex have been found to be produced in species such as *Sergentomyia minuta* and *Sergentomyia fallax*[22], their role in pheromone communication or other biological functions remains unclear. On the other hand, studies have presented behavioral evidence for the presence of sex pheromones in *Phlebotomus papatasi*, without identifying their pheromone structures [23,24].

Terpene compounds are one of the largest groups of natural products that have significant roles as repellents or attractants to many organisms [25]. They are derived from a five-carbon precursor isopentenyl diphosphate (IPP) and its isomer dimethylallyl diphosphate (DMAPP) [26]. These compounds are produced by a cellular metabolic pathway, the mevalonate route or mevalonate dependent pathway (MAD) [27], which is present in higher eukaryotes and many bacteria. Alternatively, IPP and DMAPP can also be produced through a different pathway, the methylerithritol phosphate pathway (MEP), also known as the deoxysylulose phosphate pathway (DOXP), which operates in many bacteria, plant chloroplast, and some eukaryotic parasites [28].

The MAD route involves a series of enzymatic reactions whose rate-limiting step is the reduction of the 3-hydroxy-3-methylglutaryl-CoA (HMG-CoA) to a mevalonic acid catalyzed by the enzyme 3-hydroxy-3-methylglutaryl-CoA reductase (HMG-R) [29]. Therefore HMG-R is considered the rate-limiting enzyme of this route. In insects, several studies of the mavalonate pathway have been motivated by its role in providing important bioactive molecules such as juvenile hormones [30] and aggregation pheromones (reviewed in [31]).

Following the mevalonate pathway, the two C_5_ isomeric products (IPP and DMAPP) are condensed into the monoterpe precursor (C_10_), geranyl diphosphate (GPP), catalyzed by the geranyl diphosphate synthase (GPPS). Further condensation of the C_5_ units with the GPP molecule convert into farnesyl diphosphate (FPP) (C_15_) and geranylgeranyl pyrophosphate (GGPP) (C_20_), the precursors of sesqui- and diterpene compounds among others (reviewed in [32]). These reactions are catalyzed by farnesyl diphosphate synthase (FPPS) and geranyl geranyl diphosphate synthase (GPPS), respectively, also known as short-chain prenyltransferases [33]. By applying molecular methods, these enzymes have been sequenced in several insects [34-38], some of which were directly involved in pheromone production [39,40]. Based on this, it is conceivable to hypothesize that pheromone biosynthesis in *L. longipalpis* involves the mevalonate pathway as well as prenyltransferase activity.

Molecular technologies allow the exploration of biosynthetic pathways by recognizing the expression of their enzymatic machineries [41]. This method has been successfully employed in recent studies that have analyzed enzymes in pheromone production of moths [42,43] and diterpene secretion in termites [44]. Additionally, the construction and sequencing of cDNA libraries for *L. longipalpis* has been used to obtain genetic profiles from whole insects [45] as well as from separate tissues such as salivary glands [46], midgut [47,48] and more recently, the male reproductive organ [49].

Besides the chemical identity of pheromones, there is no information about the molecular basis of pheromone production in *L. longipalpis.* In this respect, the main objective of this study was to identify the genes expressed in the pheromone gland and flanking tissues of one *L. longipalpis* population and consequently generate the first sequence catalogue for a specialized tissue where the pheromone (S)-9-methylgermacrene-B is produced. For this purpose, we constructed a cDNA library from three abdominal segments, the 3^rd^, 4^th^ (containing the pheromone gland) and 5^th^ tergites. Analyses of the expressed sequence tags (ESTs) from these segments allowed us to compare their genetic profiles and expand the spectrum of molecules possibly associated with the terpenoid pheromone. In addition, this source can also serve as a basis for future molecular research on pheromone biosynthesis and contribute to the understanding of other aspects of this insect’s biology.

## Methods

### Insects

A *L. longipalpis* (Diptera: Psychodidae) one-spot population was collected from Gruta da Lapinha, Municipality of Lagoa Santa Minas Gerais, Brazil (19° 38S; 43° 53 W). This population was colonized according to [50] at a temperature of 26°C, 80% relative humidity, and a 12:12 LD photoperiod. We used sand flies from the 1^st^ to 7^th^ generations after checking for their (S)-9-methylgermacrene-B production according to the procedures indicated in [17]. This population was maintained at the Laboratorio de Bioquímica e Fisiologia de Insetos at the Instituto Oswaldo Cruz, Rio de Janeiro, Brazil. A permit for sand fly collection was obtained from the Brazilian Ministry of Environment (SISBIO#26066-1). Tissues were extracted from male sand flies fed on a 70% sucrose solution for up to 7 days post-emergence.

### Dissections and RNA preparations

**Figure 1:**
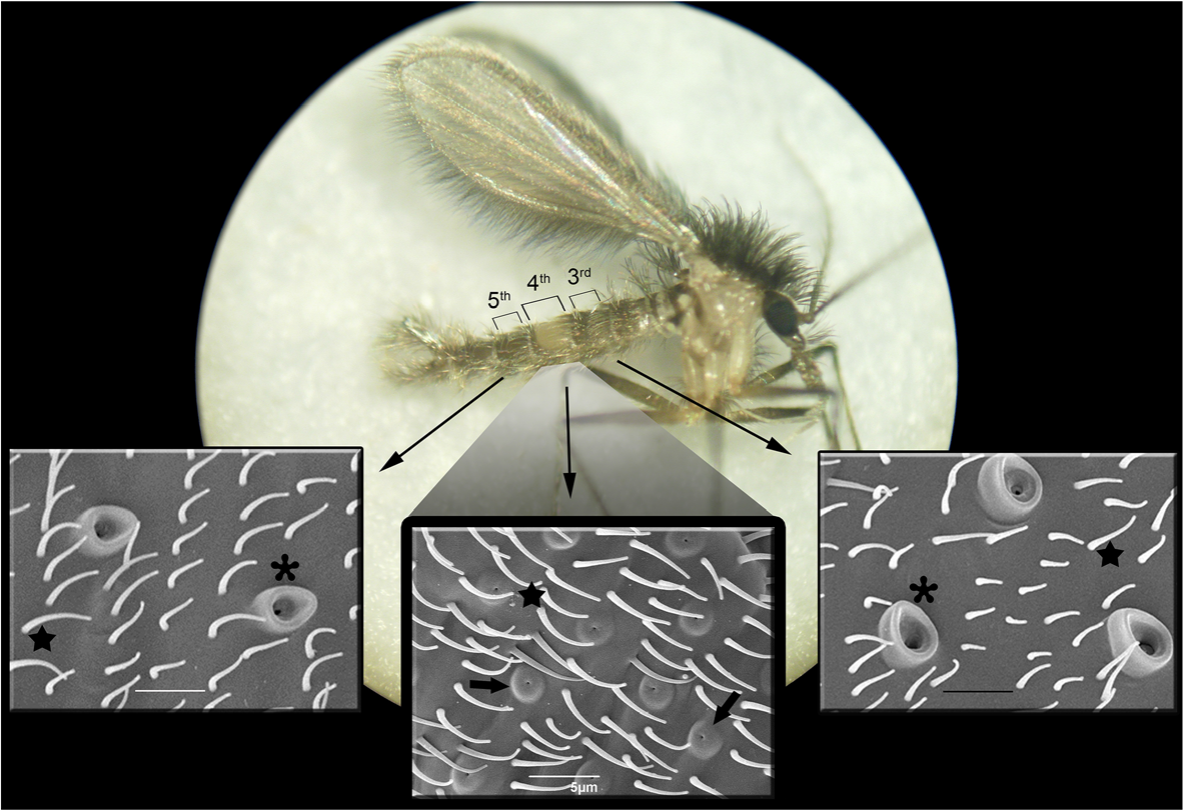
**Tissues of *L. longipalpis *male included in this study.** The stereoscope image of a *L. longipalpis* male illustrates the three abdominal segments (3^rd^, 4^th^ and 5^th^) analyzed. Characteristics of these segments are shown by scanning electron microscopy. Pheromone disseminating structures, which are present in the 4^th^ abdominal segment are indicated by the arrowhead. Macrotrichias present in the 3^rd^ and 5^th^ segment are indicated by the asterisks, and microtrichias, which are present in all abdominal segments, are indicated by the star.

Three different tissues were carefully dissected, the 4^th^ abdominal segment (LL-phg) containing the pheromone gland, as well as the 3^rd^ (LL3-seg) and 5^th^ (LL5-seg) abdominal tergites (Figure 1). After removing the midgut, the dissected segments were thoroughly washed in sterilized 0.9% saline solution in order to minimize any risk of contamination. One hundred units of the 3^rd^ abdominal segment were pooled together and preserved in an RNA preservation solution (RNAlater/Ambion) to obtain 200 ng of mRNA, the mRNA quantity used in [51]. The same procedure was repeated for each of the tissues analyzed. Messenger RNA from each tissue was purified with the Micro-FastTrack mRNA isolation kit (Invitrogen, San Diego, CA, USA) and kept at −80°C.

### Library construction and DNA sequencing

Following [47,51,52], a full-length, enriched, directionally cloned cDNA library was generated for each abdominal segment using the SMART (***S****witching****M****echanism****A****t 5*^*′*^*end of****R****NA****T****ranscript*) cDNA library construction kit (Clontech Laboratories, Montana View, CA). This technique uses oligoribonucleotide (SMART IV) to attach an identical sequence at the 5^′^end of each reverse-transcribed cDNA strand. This sequence is then utilized in subsequent PCR reactions and restriction digests.

First-strand cDNA was synthesized using PowerScript reverse transcriptase at 42°C for 1 h in the presence of SMART IV and CDS III (3^′^) primers. Second-strand synthesis was performed by a long-distance PCR-based protocol using Advantage Taq polymerase (Clontech) mix in the presence of the 5^′^ PCR prime and the CDS III (3^′^) primer. These two primers create *SfiI* A and B restriction enzyme sites at the ends of the nascent cDNA. Double-strand cDNA synthesis was carried out on a Perkin Elmer 9700 Thermal cycler (Perkin Elmer Corp., Foster City, CA, USA) in the following conditions: 95°C for 1 min, 19 cycles of 95°C for 10 s, and 68°C for 6 min. Double-stranded cDNA was immediately treated with proteinase K (0.8 μg/ml) at 45°C for 20 min, and the enzyme was removed by ultrafiltration though a Microcon YM-100 centrifugal filter device (Amicon Inc., Beverly, California, USA). The cleaned, double-stranded cDNA was then digested with *SfiI* at 50°C for 2 h, followed by size fractionation on a ChromaSpin-400 column (Clontech Laboratories, Mountain View,CA) into small (S), medium (M), and large (L) transcripts. The profile of the fractions was checked on a 1.1% agarose gel, and fractions containing cDNAs of more than 400 bp were pooled and concentrated using a Microcon YM-100.

The cDNA was ligated into the λ TripIEx2 vector (Clontech), and the resulting ligation reaction was packed using the Gigapack® Gold III Plus packaging extract (Stratagene, La Jolla, California, USA) according to the manufacturer’s instructions. The package library was plated by infecting log-phase XL1-Blue *Escherichia coli* cells (Contech). The percentage of recombinant clones was determined by blue-white selection screening on LB plates containing X-gal/IPTG. Recombinant plaques were selected and picked using a sterilized wooden stick and placed into 75 μL of ultrapure water in a 96-well v-bottom plate. The plates were covered and placed on a gyrating shaker for 30 min at room temperature. The phage suspension was either immediately used for PCR or stored at 4°C for future use. The inserts were amplified using specific library vector primers, PT2F1 (5^′^-AAGTACTCTAGCAATTGTGAGC-3^′^) and PT2R1 (5^′^-CTCTTCGCTACGCCAGCTG-3^′^), flanking the inserted cDNA. The PCR conditions were: 1 cycle of 75°C for 3 min; 1 cycle of 94°C for 3 min; 33 cycles of 94°C for 1 min, 49°C for 1 min, 72°C for 2 min, and 72°C for 7 min. We analyzed the quality of the PCR products by running 5 μL of the sample on 1.1% agarose gel and revealing them with SYBR Green.

In order to clean off salts, dNTPs, and primers, the PCR products were washed with ultrapure water three times using ExcelaPure 96-well UF PCR purification plates (Edge Biosystems, Gaithersburg, MD). Approximately 200–250 ng of each PCR product was then transferred to Thermo-Fast 96-well PCR plates (ABgene Corp, Epson, Surrey, UK) and shipped for sequencing to the Rocky Mountain Laboratories Genomics Unit as described in [52] along with the PT2F3 (5^′^-TCTCGGGAAGCGCGCCATTGT-3^′^) primer. Sequencing reactions were prepared according to the manufacturer’s protocol (BigDye®Terminator, Applied Biosystems, Inc.) by adding 1 μL ABI BigDye® Terminator ready reaction mix v3.1 (P/N 4336921), 1.5 μL 5x ABI sequencing buffer (P/N 4336699), and 3.5 μL of water for a final volume of 10 μL. Cycle sequencing was performed at 96°C for 10 sec, 50°C for 5 sec, 60°C for 4 min for 27 cycles on either a Bio-Rad Tetrad 2 (Bio-Rad Laboratories, Hercules, CA) or ABI 9700 thermal cycler (Applied Biosystems, Inc.). Fluorescently labeled extension products were purified following Applied Biosystems’ BigDye® XTerminator™ purification protocol and subsequently processed on an ABI 3730xL DNA Analyzer (Applied Biosystems, Inc.). Fluorescently labeled extension products were purified following the Applied Biosystems BigDye® XTerminator™ purification protocol and subsequently processed on an ABI 3730xL DNA Analyzer (Applied Biosystems, Inc.).

### Bioinformatics analyses

Primers or vector sequences were removed from the expressed sequence tags (EST) and then assembled into contigs using a CAP assembly program [53]. As previously described, BLAST packages and ClustalW software were used to compare and align sequences, respectively [54]. The BLASTtx algorithms were performed for functional annotation of each transcript searching against the NCBInr protein and Gene Ontology databases [55]. Conserved protein domains were predicted using reverse position specific BLAST (rpsBLAST), which searches for matches in the conserved domain database CDD [56] as well as the Pfam [57], SMART [58], and KOG [59] databases. Subsets of several organism proteomes available in the NCBI, ENSEMBL, or VectorBase were also searched. Phylogenetic analysis and statistical neighbor-joining bootstrap tests were performed using MEGA package [60]. The presence of a signal peptide was investigated using the SignalP server. Beginning with methionine found in the first 300 predicted amino acids, expressed sequence tag (EST) segments were examined every three-frame translations. Protein O-glycosylation sites were predicted using the NetOGlyc 26 software. Statistical significance in the number of transcripts per contigs, between tissues, was analyzed using a Chi-square test.

## Results and discussion

### Sequences overview

In total 3,547 phage plaques were randomly selected and sequenced, of which approximately 1,200 came from each of the three abdominal segments and were sequenced separately. After Sanger sequencing, a total of 2,502 high quality sequences were obtained. From these sequences, 1,387 clusters or contiguous sequences were assembled, of which 1,273 were represented by a single EST or singleton (Table 1). We use the term “cluster” or “contig” to refer to consensus sequences and singletons from this point on. A putative function was assigned to each contig based on the sequence homology to the databases mentioned in the methods section.

**Table 1:** **Summary of the sequence results from *L. longipalpis *pheromone gland and adjacent tissues**

**All sequences**	
Total sequence analyzed	3547
High quality sequences	2502
Contigs (clusters + singletons)	1387
Singletons	1273
Number of sequences assembled	1229
Contigs with blast match score of Eval < 10-5	751
Contigs with blast match score of Eval > 10-5	636

Sequences of most of the contigs (751) had a significant BLAST match (E-val < 10E-5) according to all reference databases, whereas 636 contigs, mostly singletons, presented low or no homology to the consulted databases. A large portion of the low homology transcripts may represent untranslated regions of genes or novel *L. longipalpis*-specific gene products.

**Figure 2:**
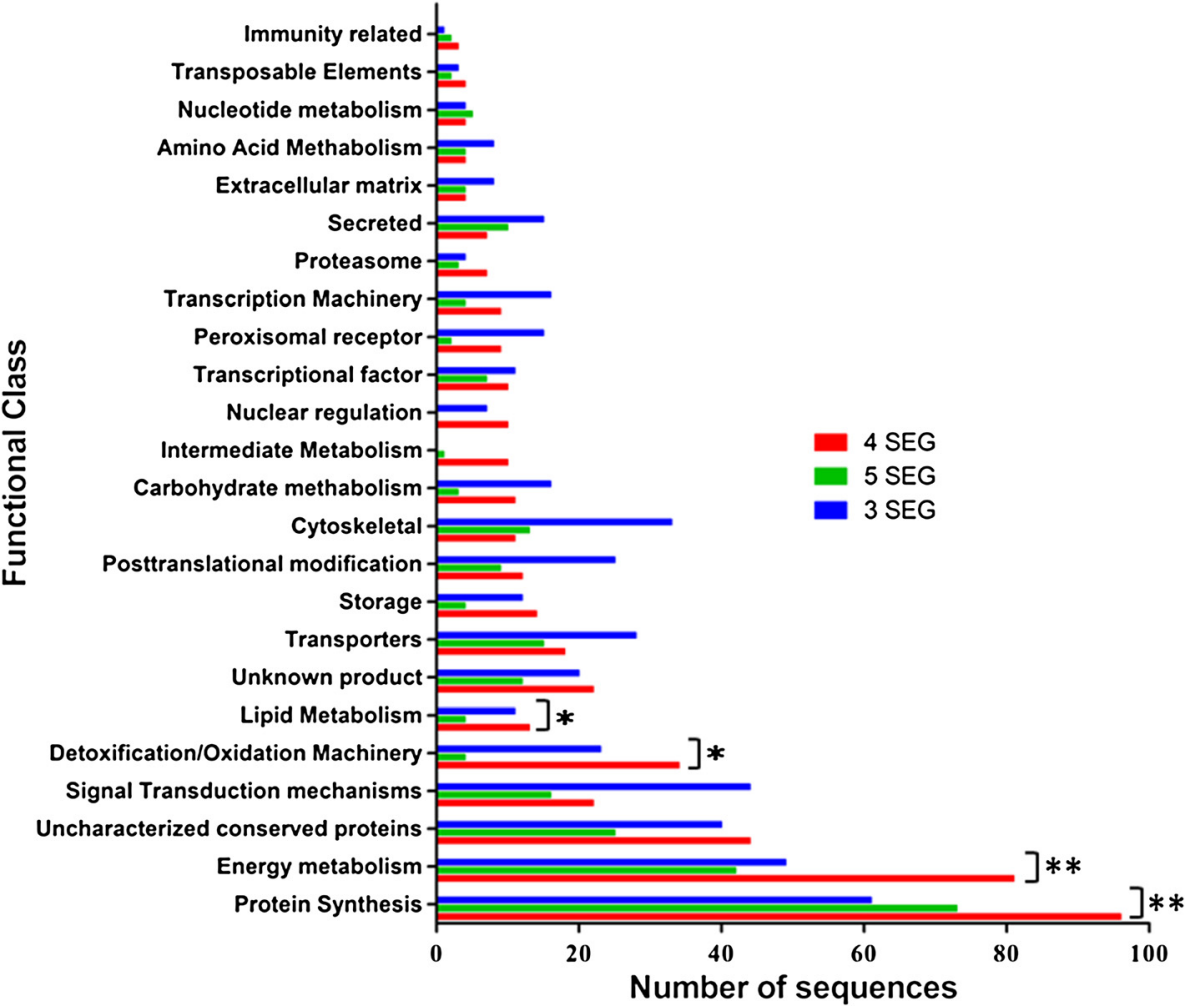
**Distribution of the number of ESTs with homology to previously described proteins grouped into functional classes from the three tissues: LL4SEG (4**^**th **^**segment containing the pheromone gland), LL3SEG (3**^**rd **^**abdominal segment) and LL5SEG (5**^**th **^**abdominal segment).** Functional classification of the transcripts was based on their sequence similarity to the proteins in the reference databases. A Chi-square test was performed for each EST tissue group across the functional categories to examine the differences between tissues. The double-asterisks represents functional classes where both differences in the number of ESTs were statistically significant (4^th^ and 3^rd^; 4^th^ and 5^th^) and the single asterisk represents functional classes where only one difference in the number of ESTs was statistically significant.

Each contig was assigned a possible function and grouped into functional categories according to the sequence homology to molecules identified by the BLAST results (E-value of less than 10E-5) from the NCBI non-redundant protein, the Gene Ontology and the conserved domain databases. Figure 2 shows the sequence abundance compared by tissue of origin across each functional category. This comparison was made after summing the contig sequences within each functional category for each tissue.

Although this histogram only shows the molecules identified by the sequence homology, it may approximately describe the biochemical characteristics of each tissue. We found more ESTs on the 4^th^ abdominal segment than the 3^rd^ and 5^th^ segments in some functional categories. For example, in the protein synthesis functional class we observed an increase of 35% in the number of ESTs on the 4^th^ segment relative to the 3^rd^, and an increase of 31% on the same segment relative to the 5^th^. These differences were statistically significant (4^th^ and 3^rd^ segments, χ^2^== 7.8, *p-*value = 5.E-03; 4^th^ and 5^th^ segments, χ^2^ = 3.13, *p-*value = 8.E-02). Similarly, in the energy metabolism functional class the greater number of ESTs found on the 4^th^ abdominal segment relative to the 3^rd^ and 5^th^ segments were also statistically significant (4^th^ seg and 3^rd^ seg χ^2^ = 7.88 and p = 5E-03; 4^th^seg and 5^th^seg χ^2^ = 12.37 and p = 4E-04). This is interesting considering greater production of proteins in the 4^th^ abdominal segment may be related to the pheromone biosynthetic machinery and the consequent energy cost. We also observed the same pattern of a greater number of ESTs on the 4^th^ abdominal segment for the lipid metabolism functional class, though the difference was statistically significant between the 4^th^ and 5^th^ segments only (4^th^ seg and 3^rd^ seg, χ^2^ = 0,17, *p* = 6,8E-01; 4^th^ seg and 5^th^ seg, χ^2^ = 20,83 *p =* 5.E-06). Similarly, we also observed more ESTs of the detoxification/oxidation functional class on the 4^th^ abdominal segment than the 3^rd^ or 5^th^ segments where the difference was statistically significant with the 5^th^ segment only (4^th^ seg and 3^rd^ seg, χ^2^ = 2,12, *p* = 1E-01; 4^th^ seg and 5^th^ seg, χ^2^ = 23,68 *p =* 1.E-06). Figure 2 highlights these functional classes and differences. These are not surprising findings considering the lipid metabolism and detoxification/oxidation machinery functional classes may include genes potentially associated to pheromone biosynthesis. The assembled contigs and BLAST results described in this paper are available in the hyperlinked Additional file 1.

### Transcripts possibly representing enzymes of the classical isoprenoid pathway

**Figure 3:**
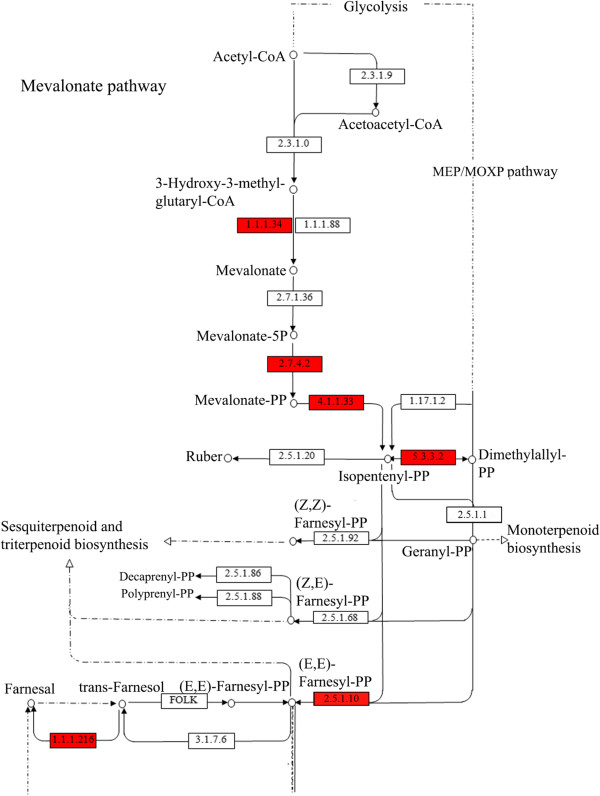
**Scheme of the classical isoprenoid pathway exhibiting enzymes for which transcripts have been identified.** Enzymes for which transcripts have been identified in the *L. longipalpis* pheromone gland only are highlighted in red.

In looking further into the group of sequences of the lipid metabolism and detoxification/oxidation functional class, we identified molecules grouped into 10 contigs potentially representing four of the seven enzymes of the mevalonate pathway. Additionally, we also found sequences similar to farnesyl diphosphate synthase (intermediate metabolism functional class) and NADP + dependent farnesol dehydrogenase of the isoprenoid pathway (Table 2). All these potentially isoprenoid-pathway related sequences were found on the 4^th^ abdominal segment only. These were not such surprising findings considering, as observed in other insects, the site where terpenoid production occurs activates the mevalonate pathway genes [44,61,62]. Figure 3 shows the classical isoprenoid biosynthetic pathway adapted from the 00900 interactive map of the Kyoto Encyclopedia of genes and genomes KEGG [63]. Enzymes for which transcripts have been identified are highlighted in the red boxes of the same figure.

**Table 2:** **Putative *L. longipalpis *pheromone gland-associated proteins; best matched results with corresponding E values from BLAST inquiries of a GenBank derived non-redundant protein database (NR) and Swiss protein database**

**Assembled contig**	**N° and origin of sequences**			**Best match**	**Database**	**E-val**	**Species of best match**
	**LL-4seg**	**LL-5seg**	**LL-3seg**				
LLphg-contig_400	1	0	0	HMG-R (gi|157122019)	NR	4.00E-33	*Aedes aegypti*
LLphg-contig_215	1	0	0	HMG-R (gi|170035703)	NR	3.00E-89	*Culex quinquefasciatus*
LLphg-contig_437	1	0	0	PMK (gi|146424700)	NR	2.00E-54	*Bombyx mori*
LLphg-contig_397	1	0	0	DMD (gi|91078238)	NR	6.00E-03	*Tribolium castaneum*
LLphg-contig_53	3	0	0	IDI (gi|157112433)	NR	2.00E-68	*Aedes aegypti*
LLphg-contig_54	3	0	0	IDI (gi|157112433)	NR	2.00E-68	*Aedes aegypti*
LLphg-contig_55	2	0	0	IDI (gi|157112433)	NR	4.00E-69	*Aedes aegypti*
LLphg-contig_56	1	0	0	IDI (sp|O35760|IDI1_RAT)	SWIISS prot.	2.00E-16	*Rattus norvegicus*
LLphg-contig_117	3	0	0	IDI (gi|157112433)	NR-LIGHT prot.	7.00E-70	*Aedes aegypti*
LLphg-contig_118	1	0	0	IDI (gi|157112433)	NR-LIGHT prot.	3.00E-65	*Aedes aegypti*
LLphg-contig_93	3	0	0	FPPS (sp|P05369|FPPS_RAT)	SWIISS prot.	6.00E-35	*Rattus norvegicus*
LLphg-contig_94	2	0	0	FPPS (sp|Q920E5|FPPS_MOUSE)	SWIISS prot.	3.00E-23	*Mus musculus*
LLphg-contig_257	1	0	0	FPPS (gi|17137582)	NR	2.00E-34	*synthetic construct*
LLphg-contig_314	1	0	0	FDH (gi|282934969)	NR	2.00E-70	*Aedes aegypti*
LLphg-contig_333	1	0	0	FDH (gi|282934969)	NR	3.00E-72	*Tribolium castaneum*

HMG-R: 3-hydroxy-3-methylglutaryl-coenzyme A reductase.

PMK: Phosphomevalonate kinase.

DMD: Diphosphomevalonate decarboxylase.

IDI: Isopentenyl-diphosphate delta isomerase.

FPPS: Farnesyl diphosphate synthase.

FDH: NADP + −dependent farnesol dehydrogenase 2.

### 3-hydroxy-3-methyl-glutaryl-CoA reductase – HMG-R (EC 1.1.1.34)

**Figure 4:**
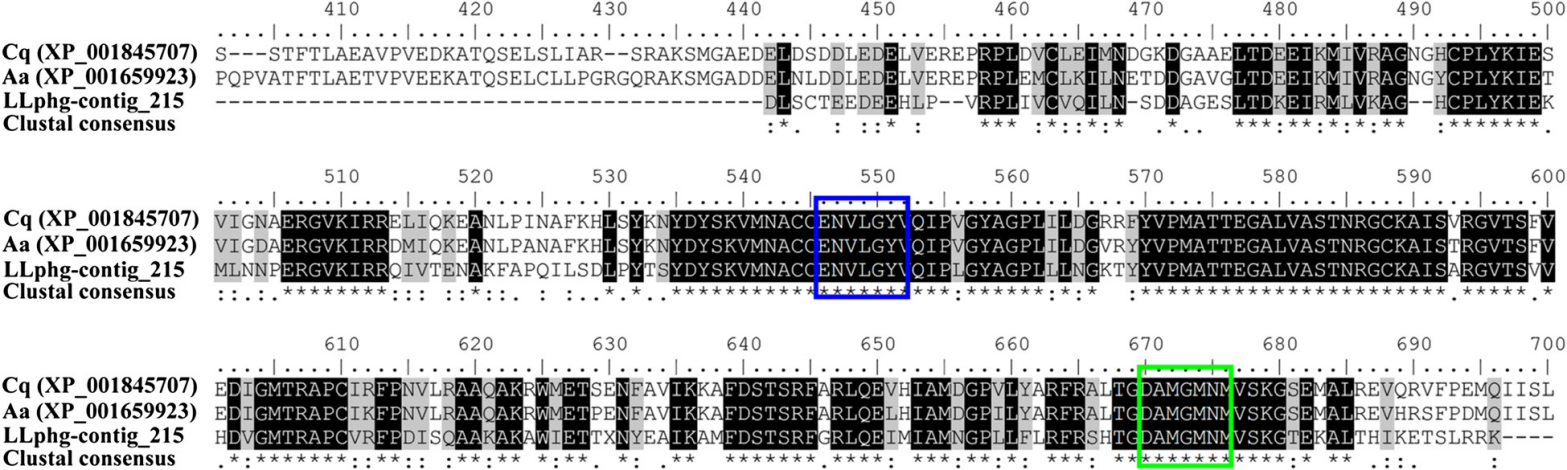
**3-hydroxy-3-methyl-glutaryl-CoA reductase (HMG-R) sequence analysis.** Sequence alignment of *Culex quinquefasciatus* (Cq), *Aedes aegypti* (Aa), and *L. longipalpis* (LLphg-contig_215) HMG-R. Identical residues are highlighted in black and similar residues highlighted in gray (>70%). The illustrated sequences represent a subset of this alignment and include residues that extend from positions four hundred to seven hundred. The predicted HMG-CoA binding motif (ENVIG) is shown in the blue box and the predicted NADP(H) binding motif (DAMGXN) is shown in the green box. The accession numbers of the sequences used are between parentheses.

We identified sequences grouped in two clusters (contigs 215 and 400) potentially encoding 3-hydroxy-3-methylglutaryl-CoA reductase (HMG-R). This enzyme has two known forms, Class I and Class II, which are present in eukaryotes and prokaryotes catalyzing the synthesis of mevalonate [64]. Although the sequences found in our library seem to be incomplete, they presented high homology to the HMG-Rs described in other insects such as *Culex quinquefasciatus* and *Aedes aegypti* (See Additional file 1 for BLAST results). They putatively encode part of the C-terminal portion of the enzyme, which is a highly conserved region containing the catalytic domain [65]. The alignment of the HMG-R C-terminal sequence of the longest transcript (contig 215) and the homologous sequences of other insects is shown in Figure 4. The conserved motifs common to HMG-R class I and II [64] are also displayed in the same figure. These motifs include the ENVIG sequence, which has a role in the HMG-CoA binding, and the DAMGXN motif, which has a role in the NADP(H) binding. The LLphg-contig_400 sequence was not used in this analysis due to its incompleteness. The differentiated expression for the gene encoding *HMG-R* and its links to terpenoid pheromone production have been reported in various coleopteran species such as *Anthonomus grandis*[66], *Ips paraconfusus*[67], *Ips pini*[68] and *Dendroctonus jeffreyi*[69,70]*.*

### Phosphomevalonate kinase - PMK (EC 2.7.4.2)

**Figure 5:**
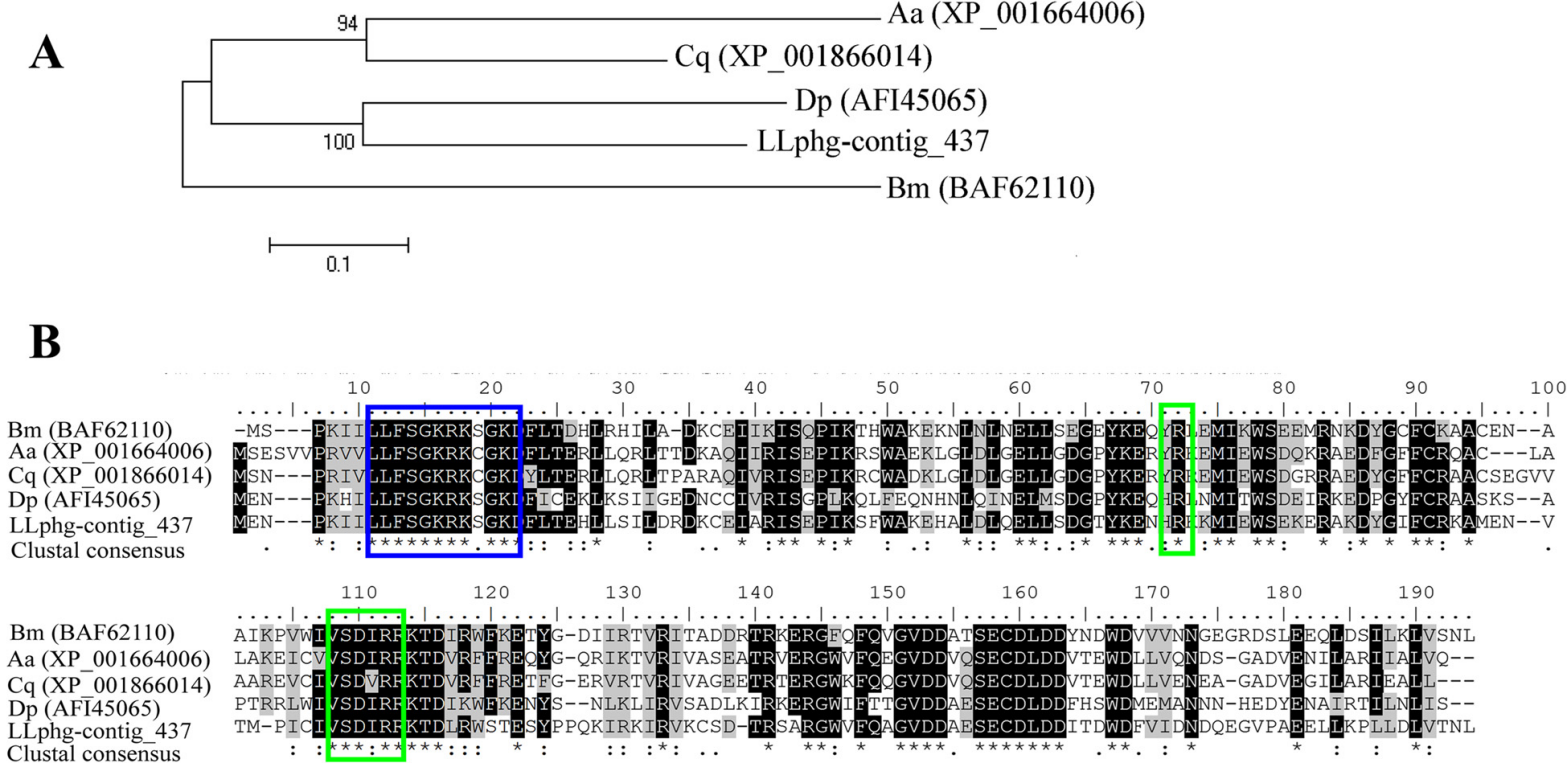
**Phosphomevalonate kinase (PMK) sequence analysis.** (**A**) Neighbor-joining tree of putative PMK from the *L. longipalpis* pheromone gland (LLphg-contig_437) *Aedes aegypti* (Aa), *Culex quinquefasciatus* (Cq), *Dendroctonus pondersae* (Dp) and *Bombix mori* (Bm). The accession numbers of the sequences used are between parentheses and node support is indicated by the bootstrap values. (**B**) Sequence alignment of PMK sequences. Identical residues are highlighted in black and similar residues highlighted in gray (>70%). Portions of the N-terminal region of these putative proteins with the phosphate binding loop is shown in the blue box and the putative mevalonate 5-phosphate binding site in the green boxes.

A unique phosphomevalonate kinase transcript (contig 437) was identified with a predicted peptide sequence of 187 amino acids. The role of this enzyme is to catalyze the reversible reaction of mevalonate-5-phosphate and ATP to produce mevalonate-5-diphosphate as well as ADP. Sequence searches against the consulted databases presented the highest similarity score to the PMK of *Aedes aegypti* (XP_001664006), *Culex quinquefasciatus* (XP_001866014) and to the PMK found in *Bombix mori* (BAF62110). Figure 5A also exhibits the comparison of our sequences to the PMK sequence found in the specialized tissues for pheromone production of *Dendroctonus pondesorase*[61]. Protein domains directly involved in mevalonate 5-phosphate phosphorylation [71,72] and ATP binding [73] were recognized in the predicted amino acid sequence of PMK (Figure 5B).

### Diphosphomevalonate decarboxylase – DMD (EC 4.1.1.33)

From contig 397 we identified a short cDNA fragment having the putative domain of diphosphomevalonate decarboxylase according to the KOG database. Despite its incompleteness, we have included this sequence in our analysis considering it may be used in future studies. This enzyme, also known as mevalonate diphosphate decarboxylase (MDD) or mevalonate pyrophosphate decarboxylase (MPD) performs the crucial step in isoprenoids biosynthesis [74] catalyzing the ATP dependent decarboxylation of the mevalonate 5-diphosphate (MDP) to yield isopentenyl diphosphate (IPP) [75]. The transcript for this enzyme was also described in the midgut of male *Ips pini*, where monoterpenoid pheromone production occurs [76].

### Isopentenyl diphosphate delta isomerase – IDI (EC 5.3.3.2)

**Figure 6:**
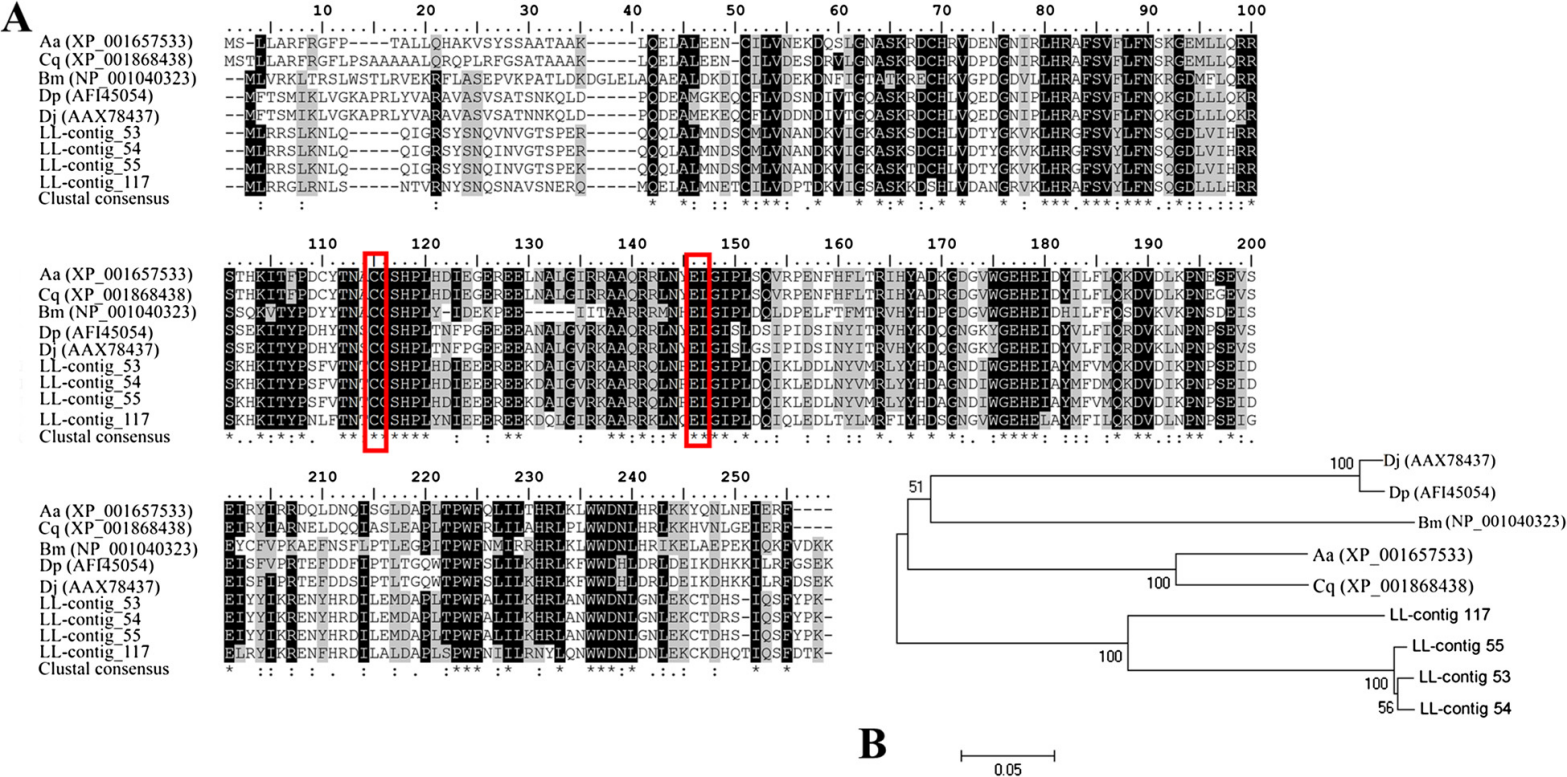
**Isopenteyl diphosphate isomerase (IDI) sequence analysis.** (**A**) Sequence alignment of IDIs from *L. longipalpis* (contigs 53, 54, 55 and 117) to reference sequences. Identical residues are highlighted in black and similar residues highlighted in gray (>70%). (**B**) Neighbor-joining tree of IDI full transcripts of *L. longipalpis* pheromone gland (contigs 53, 54, 55 and 117), *Dendroctonus jeffreyi* (Dj), *Dendroctonus ponderosae* (Dp), *Bombix mori* (Bm), *Culex Aedes aegypti* (Aa) and *quinquefasciatus* (Cq). The accession numbers of the sequences used are between parentheses and the node support indicated by the bootstrap values.

This enzyme catalyzes an essential activation in the isoprenoid biosynthetic pathway by converting isopentenyl diphosphate isomerase (IPP) to its electrophilic allylic isomer dimethylallyl diphosphate (DMAPP). IPP and DMAPP are the assembly blocks of isoprenoid compounds. We identified thirteen sequences grouped into six contigs possibly representing IDI. Contrary to the singletons (contig 56 and 118), the remaining contigs were assembled using more than one sequence. Contigs number 53, 54, and 117 were assembled with three sequences each, while contig 55 resulted from two sequences. The longest transcripts accessed from our library were compared to isopentenyl diphosphate delta isomerase found in the genome of *Aedes aegypti* (XP_001657533), *Culex quinquefasciatus* (XP_001868438) and expressed in the corpora allata of *Bombix mori* (NP_001040323.2). We also compared our transcripts to IDI sequences identified in coleopteran insects such as *Dendroctonus jeffreyi* (AAX78437) and *Dendroctunus ponderosae* (AFI45054) (Figure 6). Our phylogenetic analysis separated the *L. longipalpis* IDI-like sequences into two groups (Figure 6B), which may indicate that two forms of IDI are probably occurring in this species. The catalytic residues (reviewed in [77]) are shown in Figure 6A. The same figure exhibits the high level of similarity and identity among the sequences.

### Farnesyl diphosphate synthase - FPPS (EC 2.5.1.1./2.5.1.10)

**Figure 7:**
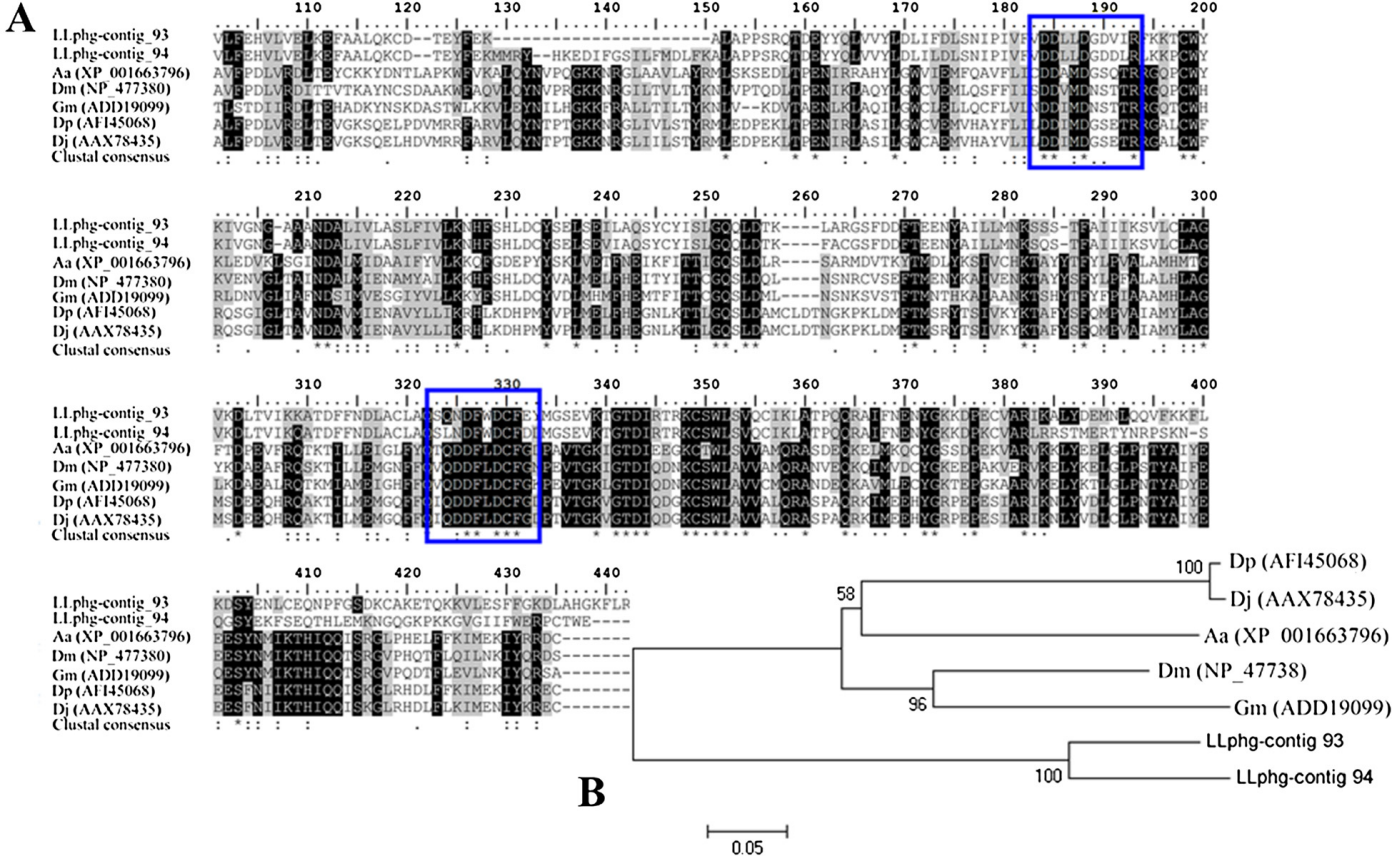
**Farnesyl diphosphate synthase (FPPS) sequence analysis.** (**A**) Subset of the sequence alignment of FPPS including residues extending from positions one hundred to four hundred forty two. Sequence alignment of the largest predicted FPPS sequences of our library (contig 94 and 93) and *Aedes aegypti* (Aa), *Drosophila melanogaster* (Dm), *Glossina morsitans morsitans* (Gm), *Dendroctonous ponderosae* (Dp), and *Dendroctonus jeffreyi* (Dj) sequences. Identical residues are highlighted in black and similar residues highlighted in gray. The aspartate-rich motifs are shown in a blue square. (**B**) Neighbor-joining tree of FPPS sequences of *L. longipalpis* pheromone gland (contigs 93 and94), *Aedes aegypti* (Aa), *Drosophila melanogaster* (Dm), *Glossina morsitans morsitans* (Gm), *Dendroctonus jeffreyi* (Dj), *Dendroctonus ponderosae* (Dp). The accession numbers of the sequences used are between parentheses and the node support indicated by the bootstrap values.

In the group of sequences classified as part of the intermediate metabolism functional class we identify molecules from three contigs (93, 94 and 257) potentially encoding farnesyl diphosphate synthase, FPPS. Enzymes catalyzing the consecutive condensation of IPP with DMAPP are known as prenyltransferases or isoprenyl diphosphate synthase [33]. Geranyl diphosphate synthase (GPPS), farnesyl diphosphate synthase (FPPS), and geranylgeranyl diphosphate synthase (GGPPS) are classified as short chain prenyltransferases and are named according to their final product: geranyl pyrophosphate (GPP) and farnesyl pyrophosphate (FPP) (reviewed in [78]). In this study we identified FPPS and no GPPS, which may indicate that FPPS is possibly involved in the synthesis of both GPP and FPP. This dual enzyme activity has been proposed and supported in independent studies [40,79-81]. Since the first characterization of FPPS in the moth *Agrotis ipsilin*[82], searches for this enzyme have been conducted in other species [35,83,84] and extended to other orders such as Coleoptera [79], Hemiptera [40,81,85], as well as Diptera [36]. Although FPPS characterizations have been traditionally associated to juvenile hormone (JH) biosynthesis (reviewed in [30]), representations of this enzyme have also been related to terpenoid-pheromone production in some insects [40,79,86]. A comparison of the *L. longipalpis* FPPS from contigs 93 and 94 to homologous sequences from other insects is shown in Figure 7. This figure also exhibits the characteristic aspartate-rich motifs, which play an essential role in substrate binding and catalysis [87,88].

### Farnesol dehydrogenase - FDH

**Figure 8:**
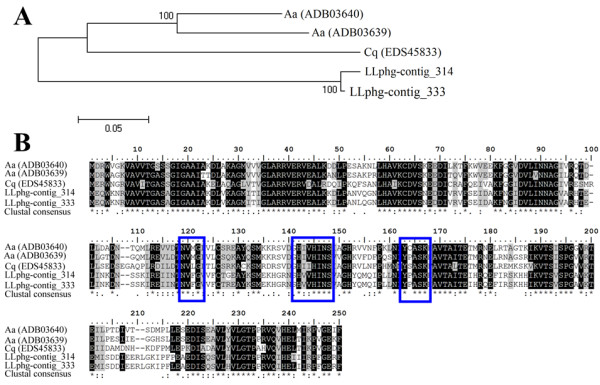
**Farnesol dehydrogenase FDH sequence analysis.** (**A**) Neighbor-joining tree and (**B**) sequence alignment of two *Lutzomyia longipalpis* farnesol dehydrogenases sequences and previously described enzymes from *Culex quinquefasciatus (Cq)* and *Aedes aegypti* (Aa). Identical residues are highlighted in black and similar residues in gray.

From contigs 314 and 333 we accessed two full-length cDNAs having 771 bp ORFs encoding predicted peptides of 247-amino acids each. The predicted protein sequences were associated to the short chain dehydrogenase/reductase (SDR) family as indicated by the SwissProt match. Additionally, by searching the NR database of the NCBI, our sequences presented a high degree of similarity to the NADP + −dependent farnesol dehydrogenase 2 (71-72%) and NADP + −dependent farnesol dehydrogenase 1 (69-70%) of *Aedes aegypti* (see Additional file 1 for blast results). This type of oxidoreductase has been previously characterized as being responsible for farnesol oxidation [89]. Figure 8 compares putative *L. longipalpis* FDH sequences with the previously described enzymes and also exhibits amino acid motifs of the enzymes’ active site [90].

This is a first study describing transcripts potentially related to enzymes making up the mevalonate pathway and prenyltransferase in *L. longipalpis.* Considering we identified these transcripts from a specialized tissue where terpenoid pheromone production occurs, these genes may be the subject of future research addressing their relationship with pheromone production.

### Transcripts associated with secreted proteins

From the sequences obtained from all three tissues containing peptide signals, an indication of secretion (Table 3), we identified molecules with predicted protein domains of odorant/pheromone binding proteins and juvenile hormone binding proteins (JHBP).

**Table 3:** **Putative *L. longipalpis *proteins; best matched results and corresponding E values from BLAST inquiries of a PFAM database**

**Assembled contig**	**N° and origin of sequences**			**Functional classification**	**Database**	**Best match**	**E-val**
	**LL-4seg**	**LL-5seg**	**LL-3seg**				
LLphg-contig_82	1	4	0	Secreted	PFAM	Haemolymph juvenile hormone binding protein	7E-049
LLphg-contig_83	1	0	0	Secreted	PFAM	Haemolymph juvenile hormone binding protein	7E-050
LLphg-contig_141	0	0	2	Secreted	PFAM	Haemolymph juvenile hormone binding protein	3E-051
LLphg-contig_142	0	0	1	Secreted	PFAM	Haemolymph juvenile hormone binding protein	9E-047
LLphg-contig_143	0	0	1	Secreted	PFAM	Haemolymph juvenile hormone binding protein	9E-052
LLphg-contig_182	0	0	2	Secreted	PFAM	Haemolymph juvenile hormone binding protein	3E-059
LLphg-contig_183	0	0	1	Secreted	PFAM	Haemolymph juvenile hormone binding protein	2E-030
LLphg-contig_230	1	0	0	Secreted	PFAM	Haemolymph juvenile hormone binding protein	1E-024
LLphg-contig_336	1	0	0	Secreted	PFAM	Haemolymph juvenile hormone binding protein	3E-020
LLphg-contig_736	0	0	1	Secreted	PFAM	Haemolymph juvenile hormone binding protein	5E-025
LLphg-contig_95	0	0	2	Secreted	PFAM	OS-D/Insect pheromone-binding family	1E-051
LLphg-contig_96	1	0	0	Secreted	PFAM	OS-D/Insect pheromone-binding family	4E-042
LLphg-contig_97	0	0	1	Secreted	PFAM	OS-D/Insect pheromone-binding family	1E-051
LLphg-contig_98	0	1	0	Secreted	PFAM	OS-D/Insect pheromone-binding family	2E-006
LLphg-contig_954	0	0	1	Secreted	PFAM	General odorant binding protein family	3E-027
LLphg-contig_1236	0	1	0	Secreted	PFAM	General odorant binding protein family	1E-027
LLphg-contig_78	2	0	0	Secreted	PFAM	General odorant binding protein family	3E-020
LLphg-contig_79	2	0	0	Secreted	PFAM	General odorant binding protein family	2E-017
LLphg-contig_80	1	0	0	Secreted	PFAM	General odorant binding protein family	6E-020
LLphg-contig_81	1	0	0	Secreted	PFAM	General odorant binding protein family	9E-020
LLphg-contig_1059	0	0	1	Secreted	PFAM	General odorant binding protein family	3E-024

### 3.1 Transcripts related to insect odorant binding protein (OBP)

Members of this family are ubiquitous in insects and are specialized in carrying small hydrophobic ligands through aqueous media [91]. According to PFAM database searches, sequences grouped in seven contigs (78, 79, 80, 81, 1059, 954 and 1236) presented conserved domains of pheromone/odor binding proteins. Additionally, sequences from contig 954 and 1236 presented similarity to the 99a OBP sequence described in *C. quinquefasciatus* (EDS45783) deposited in the NR database. Another group of sequences reunited in contigs 95, 96, and 98 also presented a high degree of similarity to proteins referred as OS-D proteins, chemosensory proteins (CSPs) or sensory appendage proteins (SAPs), based on their association with insect sensory organs [92]. Given these molecules are highly conserved across species [92], our finding led us to consider the possibility that they may also facilitate pheromone transportation between the pheromone gland and the external environment, as in moths [93-95].

### Juvenile hormone binding protein (JHBP)

Ten molecules were identified as probable haemolymph juvenile hormone binding proteins (JHBP). It is known that JHBPs transport the highly hydrophobic insect juvenile hormone (JH) from the most important site of its synthesis (the corpora allata) to target tissues [96,97]. By analogy to the role of JHs in the regulation of pheromone production in beetles, cockroaches, and moths [98-101] the possible expression of JHBP in the tissues analyzed here may represent trace evidence of a similar function in *L. longipalpis*. However, it is also possible that due to the chemical similarity between the JHs and the *L. longipalpis* sesquiterpene pheromone, the JHBP gene products might bind both JH and *L. longipalpis* pheromones.

### Transcripts related to detoxification/oxidation machinery

#### Cytochrome P450

Another interesting group of transcripts were those encoding putative cytochrome P450 enzymes. These sequences were grouped into thirteen contigs obtained from all three tissues used in this analysis (Table 4). Cytochrome P450 (CYP) comprises a large family of heme-thiolate enzymes that metabolize a wide range of endogenous and exogenous compounds. In insects, these enzymes are found in virtually all tissues [102] and catalyze the metabolism of exogenous substances and participate in the biosynthesis and degradation of juvenile hormones, ecdysteroids, and pheromones [103-105]. In our work, two P450 protein subfamilies were identified: CYP4/CYP19/CYP26 and CYP3/CYP5/CYP6/CYP9, according to the eukaryotic orthologous group database (KOG). The CYP4/CYP19/CYP26 subfamily was the most abundant and it was represented in contigs 39, 40, 41, 42, 43, 615, and 792. The occurrence of a higher abundance of transcripts for CYP4 and CYP3 P450 protein families was also recently described in a transcriptome study of different *Dendroctonus ponderosae* tissues, including the terpenoid pheromone production site [61].

**Table 4:** **Putative *L. longipalpis *proteins; best matched results and corresponding E values from BLAST inquiries of a GenBank derived non-redundant protein database (NR), KOG and Swiss prot databases**

**Assembled contig**	**N° and origin of sequences**			**Class**	**Database**	**Best match**	**E-val**
	**LL-4seg**	**LL-5seg**	**LL-3seg**				
LLphg-contig_39	4	0	0	Detox/ox	KOG	Cyt P450 CYP4/CYP19/CYP26 subfamilies	1E-048
LLphg-contig_40	0	2	2	Detox/ox	KOG	Cyt P450 CYP4/CYP19/CYP26 subfamilies	1E-063
LLphg-contig_41	0	0	3	Detox/ox	KOG	Cyt P450 CYP4/CYP19/CYP26 subfamilies	1E-106
LLphg-contig_42	1	1	3	Detox/ox	KOG	Cyt P450 CYP4/CYP19/CYP26 subfamilies	1E-108
LLphg-contig_43	0	0	1	Detox/ox	KOG	Cyt P450 CYP4/CYP19/CYP26 subfamilies	1E-044
LLphg-contig_615	1	0	0	Detox/ox	KOG	Cyt P450 CYP4/CYP19/CYP26 subfamilies	6E-034
LLphg-contig_792	0	0	1	Detox/ox	KOG	Cyt P450 CYP4/CYP19/CYP26 subfamilies	2E-052
LLphg-contig_305	1	0	0	Detox/ox	KOG	Cyt P450 CYP3/CYP5/CYP6/CYP9 subfamilies	2E-072
LLphg-contig_610	1	0	0	Detox/ox	KOG	Cyt P450 CYP3/CYP5/CYP6/CYP9 subfamilies	3E-071
LLphg-contig_915	0	0	1	Detox/ox	KOG	Cyt P450 CYP3/CYP5/CYP6/CYP9 subfamilies	1E-111
LLphg-contig_930	0	0	1	Detox/ox	KOG	Cyt P450 CYP3/CYP5/CYP6/CYP9 subfamilies	8E-083
LLphg-contig_940	0	0	1	Detox/ox	KOG	Cyt P450 CYP3/CYP5/CYP6/CYP9 subfamilies	3E-087
LLphg-contig_1067	0	0	1	Detox/ox	KOG	Cyt P450 CYP3/CYP5/CYP6/CYP9 subfamilies	2E-083
LLphg-contig_156	1	0	0	Detox/ox	NR	Glutathione S-transferase	2E-066
LLphg-contig_157	0	1	0	Detox/ox	NR	Glutathione S-transferase	3E-080
LLphg-contig_158	1	0	0	Detox/ox	NR	Glutathione S-transferase	4E-081
LLphg-contig_774	0	0	1	Detox/ox	NR	Glutathione S-transferase	1E-102
LLphg-contig_789	0	0	1	Detox/ox	NR	Glutathione S-transferase	1E-124
LLphg-contig_1066	0	0	1	Detox/ox	NR	Glutathione S-transferase	5E-087
LLphg-contig_367	1	0	0	Detox/ox	SWISSP prot.	Juvenile hormone epoxide hydrolase 1	3E-094
LLphg-contig_868	0	0	1	Detox/ox	SWISSP prot.	Juvenile hormone epoxide hydrolase 2	2E-047

### Glutathione S-transferase

Another noticeable gene involved in biotransformation is the glutatione S-transferase. We identified sequences grouped into six contigs (156, 157, 158, 774, 789, and 1066) with high similarity to glutation S-transfererase previously described in *L. longipalpis* (Table 4 and Additional file 1). Besides its important role for detoxification, especially in phytophagous insects [106], these molecules have also been described as odor degradation enzymes in some insects [107].

### Juvenile hormone epoxide hydrolase - JHEH

After consulting the SWISSP database two transcripts presented high sequence similarity to juvenile hormone epoxide hydrolase (JHEH), one obtained from the 4^th^ abdominal segment library containing the pheromone gland (contig 367), and the other from the 3^rd^ abdominal segment library (contig 868). The JHEHs together with the juvenile hormone esterase (JHE) are known as enzymes having a role in JH degradation [108].

## Conclusion

This study has confidently generated a list of transcripts expressed in the pheromone gland and adjacent tissues. Molecules associated to enzymes of the mevalonate pathway were found only in the 4^th^ abdominal segment (containing the pheromone gland). The identification of these enzymes was facilitated by the conservation of the mevalonate pathway between insect species and vertebrates [30]. However, this conservation is not present in the later steps of isoprenoid compound production, which involves several paths leading to different final products. With the exception of juvenile hormones [30], there is little available information on the biosynthesis of cyclic sesquiterpene or homosesquiterpene compounds in insects. Such advances in this field could contribute to the identification of the specialized enzymatic machinery through a classical homology inference. In this context, the unmatched groups of sequences we obtained from the 4^th^ abdominal segment constitute a useful resource for future explorations of the specialized enzymatic components of (S)-9-methylgermacrene-B production.

Furthermore, in this work we also present a set of interesting genes potentially related to the insect OBP protein family, including molecules similar to chemosensory proteins. This finding also provides an interesting subject for future studies considering its potential implication for pheromone transportation [93] or other functions [109]. This transcriptome sequence resource may also provide new molecular tools for future studies of other terpene-producing *L. longipalpis* populations. Finally, our results may also serve to identify potential targets for genetic manipulation techniques for population monitoring and control of *L. longipalpis*, considering this insect carries the parasite causing an important and neglected tropical disease, visceral leishmaniasis.

## Competing interests

The authors declare that they have no competing interests.

## Authors’ contributions

Conceived and designed the experiments: NGC, PC and RPB. Performed the experiments: NGC and JGV. Analyzed the data: NGC, JGV and JMR. Contributed reagents/materials/analysis tools: RPB, JGV and JMR. Wrote the paper: NGC, PC and RPB. All authors read and approved the final version of the manuscript.

## Supplementary Material

Additional file 1 Excel File with assembled contigs and BLAST results. Click here for file
